# Supplementary material for: Electrochemical Fingerprint Analyses Using Voltammetry and Liquid Chromatography Coupled with Multivariate Analyses for the Discrimination of Schisandra Fruits
Source: Molecules. 2024 Dec 26;30(1):48. doi: 10.3390/molecules30010048 (PMC11721992; doi:10.3390/molecules30010048)
Supplement: Supplementary file 1 [file molecules-30-00048-s001.zip › molecules-3313740-supplementary.pdf]

Supplementary Materials

Electrochemical fingerprint analyses using voltammetry and liquid chromatography coupled with multivariate analyses for the discrimination of Schisandra fruits

Koichi Machida, Akira Kotani,* Tomoya Osaki, Ayaka Kobayashi, Kazuhiro Yamamoto, Hideki Hakamata

Department of Analytical Chemistry, School of Pharmacy, Tokyo University of Pharmacy and Life Sciences, 1432-1 Horinouchi, Hachioji, Tokyo, 192-0392 Japan

* *Corresponding Author: kotani@toyaku.ac.jp*

Table S1. Cross validation of PLS-DA for the data sets obtained from the voltammetry [a]

	Component				
	1	2	3	4	5
Accuracy [b]	1	1	1	1	1
R^2 [c]	0.89303	0.89774	0.90615	0.91235	0.94035
Q^2 [d]	0.86878	0.86779	0.86578	0.86721	0.89626

[a] Model performance was evaluated using Leave-One-Out Cross-Validation (LOOCV), where each sample is used exactly once as a validation set, while all other samples are used for training. This iterative process, repeated for all n samples, provides a conservative and unbiased assessment of model performance. The performance metrics reported here represent the aggregated results across all n iterations, providing a robust estimate of the model's true generalization capability.

[b] Accuracy is an indicator of the model classification performance in the PLS-DA. For example, Accuracy = 1 at component 1 means that the probability of correctly identifying an SSF sample among Schisandra fruit samples was 100%.

[c] R^2 is the coefficient of determination. The closer the R^2 value is to 1 means the predicted model in the PLS-DA is correlates to the real model obtained from known SSF and SCF samples.

[d] Q^2 is the predictive coefficient of determination, which is an estimate of the predictive ability of the model. The closer the Q^2 value is to 1 means an unknown Schisandra fruit sample can be correctly discriminated as an SSF or SCF sample by the classifier in the PLS-DA.

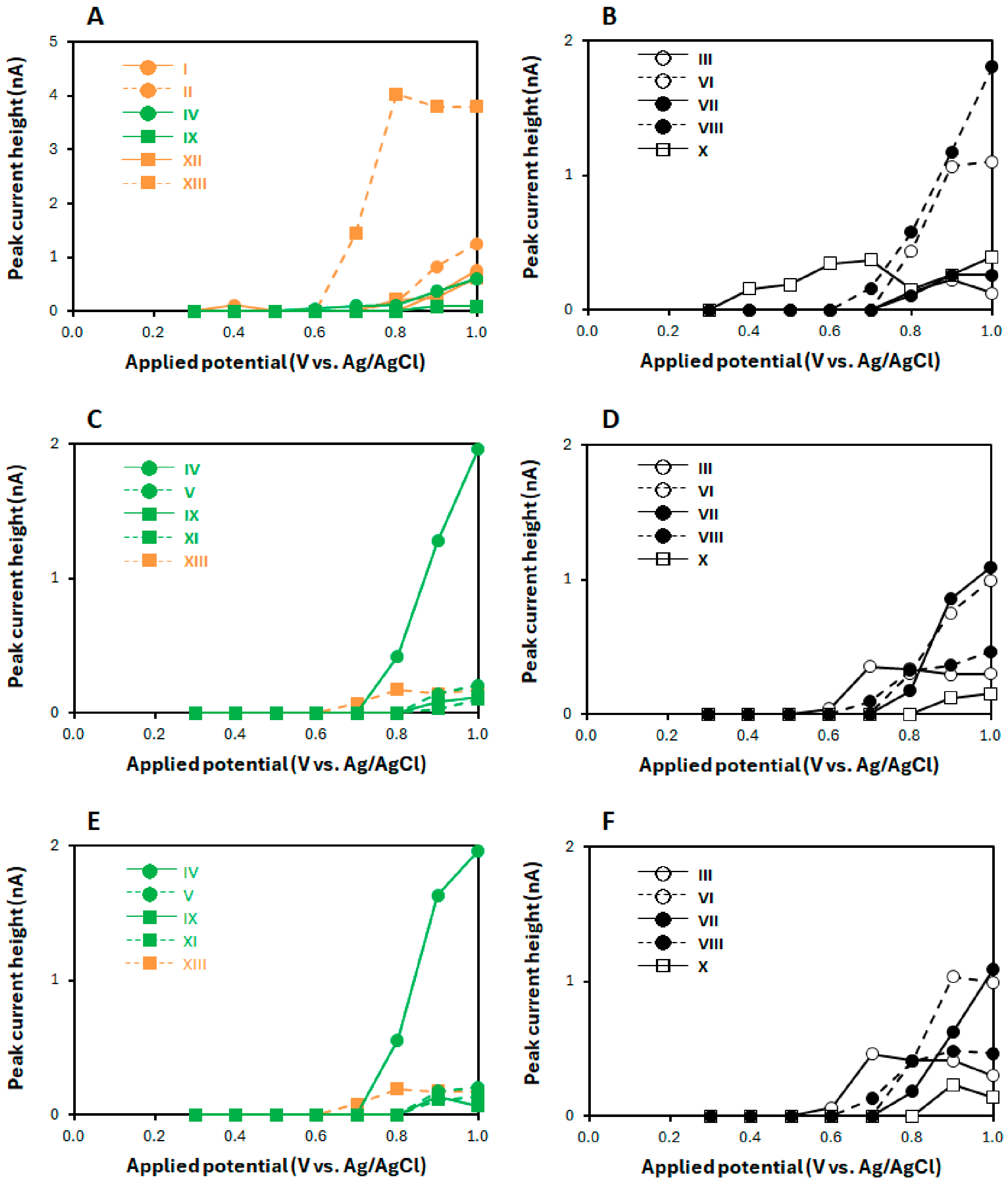

Figure S1. Hydrodynamic voltammograms of electro-active components in methanol extracts of (A, B) SSF, (C, D) SCF, and (E, F) JPSF samples by the HPLC-ECD.

Table S2 Cross validation of PLS-DA for the data sets obtained from the HPLC-ECD [a]

	Component				
	1	2	3	4	5
Accuracy [b]	1	1	1	1	1
R^2 [c]	0.95892	0.96774	0.9706	0.97328	0.97766
Q^2 [d]	0.95123	0.95429	0.94966	0.94909	0.94663

[a-d] The summaries regarding these parameters are described in the footnotes of Table S1.

Table S3 List of Schisandra fruit samples

SSF [a]		SCF [a]		JPSF	
Production region (Province)	Sample #	Production region (Province)	Sample #	Production region (Province)	Sample #
Hunan	SSF1-01	Liaoning	SCF1-01	Liaoning	JPSF1-01
	SSF1-02		SCF1-02		JPSF1-02
Henan	SSF2-01		SCF1-03		JPSF2-01
	SSF2-02		SCF1-04		
	SSF2-03		SCF1-05		
	SSF2-04		SCF1-06		
	SSF2-05		SCF1-07		
Jiangxi	SSF3-01		SCF1-08		
	SSF3-02		SCF1-09		
	SSF3-03	Hebei	SCF2-01		
Guangxi	SSF4-01		SCF2-02		
Shanxi	SSF5-01		SCF2-03		
Ningxia	SSF6-01		SCF2-04		
Guangdong	SSF7-01	Jilin	SCF3-01		
			SCF3-02		
			SCF3-03		
			SCF3-04		

[a] The SSF and SCF samples were identified by Dr. Liangmian Chen, an Associate Professor at the Institute of Chinese Materia Medica, China Academy of Chinese Medical Sciences, Beijing, China.

Summary of normalizing before the PCA and PLS-DA

The summary of normalizing is explained using the current values on a voltammogram. The current values obtained from a voltammogram are the data of time series because the currents were recorded when the applied potential was swept at a constant scan rate. Thus, the current values on a voltammogram, I_t, from data point 1 to *n* are given as follows:

$$\{I_t\} = \{I_1, I_2, I_3, I_4, \cdots, I_n\} \quad \text{(S1)}$$

First, the average value of I_t, $\bar{I}$, is calculated using Eq. (2).

$$\bar{I} = \frac{1}{n}\sum_{t=1}^{n} I_t \quad \text{(S2)}$$

The processed current values on the voltammogram by the normalizing, I'_t, from data point 1 to *n* were obtained by dividing by I_t by $\bar{I}$ as follows:

$$\{I'_t\} = \left\{\frac{I_1}{\bar{I}}, \frac{I_2}{\bar{I}}, \frac{I_3}{\bar{I}}, \frac{I_4}{\bar{I}}, \cdots, \frac{I_n}{\bar{I}}\right\} \quad \text{(S3)}$$

These processes are called normalizing in terms of multivariate analysis and the data sets of $\{I'_t\}$ obtained from the voltammograms of the Schisandra fruits samples were used to perform PCA and PLS-DA. By the normalizing in this study, the characteristic oxidation waves were recognized on the voltammograms of the Schisandra fruit samples.

Summary of autoscaling before the PCA and PLS-DA

The summary of autoscaling is explained using the current heights of characteristic target peaks I-XIII observed on the chromatogram of the Schisandra fruit samples. Figure S1(A) shows box-and-whisker charts of the current heights of target peaks obtained from chromatograms of Schisandra fruit samples. As shown in Fig. S1(A), averages and standard deviations (SDs) of the current heights are different among target peaks I-XIII before the autoscaling. By the autoscaling, the averages and SDs values of the current heights of all of the target peaks are corrected to 0 and 1, respectively. Figure S1(B) shows box-and-whisker charts of the current heights of target peaks I-XIII after the autoscaling. By the autoscaling, the systematic bias or technical variation can be reduced.

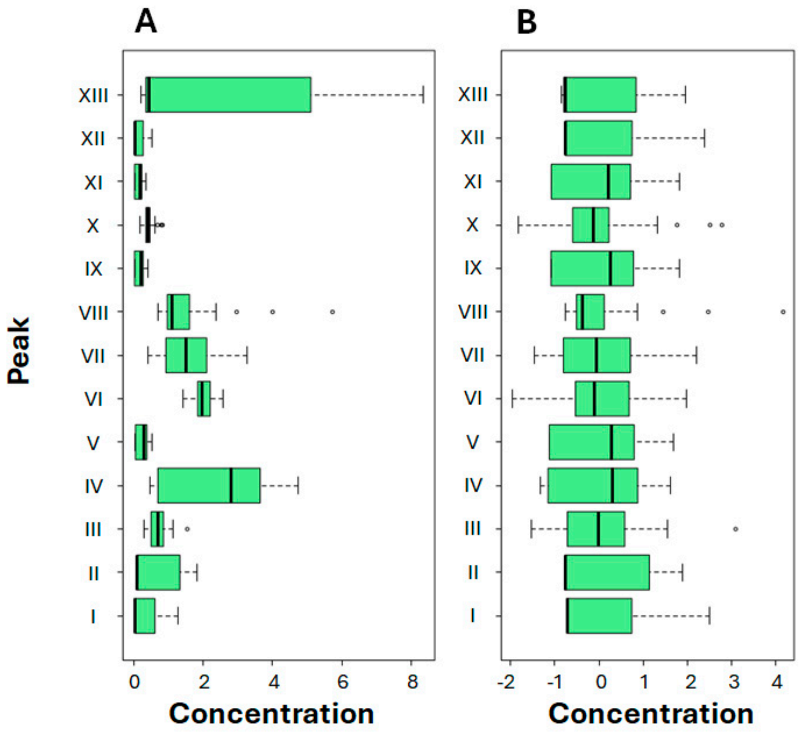

Figure S2. Box-and-whisker charts of the current heights of the target peaks I-XIII obtained from the chromatograms of the Schisandra fruit samples (A) before and (B) after autoscaling.
